# *Sarcodia suiae* Water Extract Promotes the Expression of Proinflammatory and Th1-Type Cytokines and Delay the Onset of Mortality in Cobia (*Rachycentron canadum*) During *Photobacterium damselae* subsp. *damselae* Infection

**DOI:** 10.3389/fimmu.2021.801501

**Published:** 2022-01-24

**Authors:** Po-Tsang Lee, Fan-Hua Nan, Po-Yu Chiu, Chung-Chih Tseng, Meng-Chou Lee

**Affiliations:** ^1^ Department of Aquaculture, National Taiwan Ocean University, Keelung City, Taiwan; ^2^ Institute of Medical Science and Technology, National Sun Yat-sen University, Kaohsiung City, Taiwan; ^3^ Zuoying Branch of Kaohsiung Armed Forces General Hospital, Kaohsiung City, Taiwan; ^4^ Center of Excellence for Ocean Engineering, National Taiwan Ocean University, Keelung City, Taiwan; ^5^ Center of Excellence for the Oceans, National Taiwan Ocean University, Keelung City, Taiwan

**Keywords:** immunostimulants, innate immunity, marine fish, macroalga, bacterial infection, cytokines, aquaculture

## Abstract

Cobia (*Rachycentron canadum*) is a marine fish of high economic value that grows at a fast rate. However, intensive fish farming has led to disease outbreaks in cobia cultures, which is highly costly to the industry. The impact of infectious diseases on cobia production has led to the inappropriate and increased use of chemicals and antibiotics, which negatively affects the environment and human health and promotes the spread of drug-resistant pathogens. Hence, prophylactic measurements, such as the use of immunomodulators, are required to improve the health of cultured animals against pathogens. In this study, we examined the effects of *Sarcodia suiae* water extract (SSWE) in cobia *in vitro* and *in vivo*. We found that treatment with SSWE could significantly increase the expression of cytokines (e.g., *IL-1β*, *IL-6*, *IL-10*, *IL-12*, and *TNF-α*) and chemokines (e.g., *IL-8*) in primary cultured head kidney leukocytes. Intraperitoneal injection of SSWE (20 μg/g body weight) promoted higher expression of *IL-6*, *IL-8*, *IL-10*, *IL-12*, chemokines (e.g., *CC1*), and antibodies (e.g., *IgT*) in head kidney and spleen tissues of the fish compared with other dose levels. Additionally, we describe for the second time (only after India) of the isolation of *Photobacterium damselae* subsp. *damselae* (*Phdd*) from a deadly epizootic in cage-farmed cobia. An intraperitoneal inoculation of SSWE before *Phdd* challenge showed that SSWE treatment could delay the onset of mortality of cobia. Finally, fish that received SSWE intraperitoneally before infection with *Phdd* exhibited elevated expression of Th1-type cytokines, namely, *IL-8*, *IL-12*, *TNF-α*, and *IFN-γ*. At the same time, the expression of Th2-related factors (such as *IL-10* in the head kidney, and *IgM* and *IgT* in the spleen) were lower for the fish that received SSWE instead of PBS before the *Phdd* challenge. The results indicate that SSWE treatment facilitates the induction of Th1-type cytokines in cobia to fight against *Phdd* infection and has the potential to be used as an immunostimulant and vaccine adjuvant for fish.

## Introduction

*Rachycentron canadum*, commonly known as cobia, can increase in mass by up to 6 kg a year. Cobia are known for their delicious taste and fast growth rate (1). Wild cobia are widely distributed in the warm waters outside of the Eastern Pacific Region and are found in the surrounding waters of Taiwan. Cobia is an ideal cage-cultured species (2), and in Taiwan, it is mainly produced in Penghu County, Pingtung County, and Xiaoliuqiu Island. The four leading cobia farming countries include China, Vietnam, Panama, and Taiwan, with an estimation of 53,000 metric tons in 2020 (3). However, intensive farming often leads to disease outbreaks, resulting in unstable annual outputs (4). The most common diseases for cobia are caused by bacteria, followed by parasites and viral diseases. The parasitic pathogens for cobia include *Neobenedenia* spp (5)., *Caligus* spp (6)., *Trichodinidae*, *Cryptocaryon irritans* (7), and *Amyloodinium ocellatum* (8); while viral diseases are mainly lymphatic cysts (lymphocystis virus disease) (2). The most common bacterial diseases are vibriosis (9, 10), mycobacteriosis (11), lactococcosis (12), streptococcosis (13), and photobacteriosis (mainly caused by *Photobacterium damselae* subsp. *piscicida*) (14, 15). Recently, a pathogenic strain of *P. damselae* subsp. *damselae* (*Phdd*) was isolated from a deadly epizootic in cage-farmed cobia for the first time (16), but currently there is no information regarding on *Phdd* infection to wild cobia.

Immunomodulators from natural resources are known for their environmentally friendly properties and their ease of application (17). Immunomodulators are used as prophylactic agents and are commonly exploited in aquaculture to strengthen the health and disease resistance of cultured animals (18). Algae are photosynthetic organisms found on land and in the ocean. Macroalgae (seaweeds) grow in coastal areas, and do not possess organs commonly found in terrestrial plants (19). The marine macroalga *Sarcodia suiae* is edible and moderately abundant in the coastal areas of Taiwan. Our previous study was the first to demonstrate the immunomodulatory effects of *S. suiae* water extract (SSWE) in a freshwater fish, Nile tilapia (*Oreochromis niloticus*) (20), but the immunoregulatory properties of SSWE for marine fish remain unknown.

Recently, we isolated *Phdd* from diseased cage-farmed cobia in Pingtung County; *Phdd* had never been previously reported in cultured cobia in Taiwan. As part of the present study, a preliminary virulence study suggested that *Phdd* can cause mortality in juvenile cobia, and bacteria were successfully re-isolated from the blood and tissues (e.g., liver, spleen, and head kidney) of moribund fish. Therefore, in the present study, we examined the effects of SSWE *in vitro* and *in vivo* by analyzing the transcript modulation of immune-related genes in primary head kidney cells of cobia, and in immune-related tissues from fish intraperitoneally treated with extracts. Furthermore, protective properties of SSWE were evaluated in cobia against *Phdd*. Finally, we assessed the expression of immune-related genes from fish that received SSWE intraperitoneally before infection with *Phdd*.

## Materials and Methods

### Experimental Fish

Healthy cobia (*R. canadum*) from a local farm (Pingtung County, Taiwan) were transported to the laboratory and kept in 2 m^3^ fiber-reinforced plastic (FRP) tanks with continuous fresh seawater flow (2 L/min). Commercial feed (Tairoun Products Company Ltd, Taipei, Taiwan) was given twice a day at 3% of total fish body weight. Water parameters were maintained as follows: temperature at 28 ± 2°C, pH at 8.1 ± 0.1, and dissolved oxygen above 5.0 mg/L. The animal experiments were approved by the NTOU Institutional Animal Care and Use Committee (approval number: 109045) and were performed in accordance with the animal experimentation procedure guidelines. Moribund fish that with erratic, disoriented swimming movements from bacteria challenge assays, were euthanized by using an overdose of MS222 (Sigma-Aldrich, USA).

### Preparation of SSWE

*S. suiae*, a marine Rhodophyta algal species, was obtained from a previous study (21). As described previously, a healthy adult individual with a wet weight of 5.0 ± 0.5 g was selected and pre-cultured for three days in sterile seawater (salinity at 30 ppt). *S. suiae* algae powder (1 g) was mixed with nuclease-free water (20 ml; UltraPure™ Distilled Water, Invitrogen, USA) in an Erlenmeyer flask and incubated at 40°C in a water bath (BH-130 D; YIHDER, Taiwan) for 3 h. During this period, the conical flask was shaken several times every 15–30 min to ensure uniform heating. Afterwards, the sample was cooled to room temperature and the contents were transferred into centrifuge tubes, followed by centrifugation at 10,000×*g* at 4°C for 10 min (Allegra X-30R Centrifuge; Beckman Coulter, USA). The supernatant was collected and freeze-dried using a freeze dryer (FD4.5/-50; Firstek, Taiwan). This dried extract is subsequently referred to as SSWE. Patent application in Taiwan (application number 109108939) for the extraction methods is under review.

### *In Vitro* Experiments

#### Stimulation of Primary Head Kidney Leukocytes

Head kidneys (HKs) were removed from five healthy cobia (200 ± 25 g), and an HK cell suspension was prepared using previously reported procedures (22) with slight modifications. Briefly, HK tissues were gently pressed through a 100 µm nylon mesh using a sterile spatula and incomplete media consisting of L-15 medium (Gibco, USA) supplementing with 2% (v/v) fetal bovine serum (FBS; Corning, USA), penicillin (100 U/ml)–streptomycin (100 μg/ml) (P/S) (Gibco), and 10 U/ml heparin as an anticoagulant (Sigma-Aldrich, USA). Cells were washed twice using the incomplete media, then adjusted to 1 × 10^7^ cells per well in complete growth media, distributed over 6-well plates. The complete growth media had a similar composition to the incomplete media, except the FBS ratio was increased to 10% and no heparin was added. Cells were incubated for 1, 2, and 4 h in L-15 containing 0, 0.2, and 2 μg/ml of SSWE.

### *In Vivo* Experiments

#### *In Vivo* Modulation of Transcript Levels of Immune-Related Genes in Cobia by SSWE

Fish (average weight 150–200 g) were injected intraperitoneally with PBS or PBS containing different doses of SSWE (0.2, 2, and 20 μg/g body weight (BW)). Fish (n = 3 per group) were sacrificed, and HK and spleen tissue samples were harvested at 4, 8, and 24 h post-injection. Tissues were preserved in RNA Preserve Solution (BIONOVAS, Canada) and stored at −80°C until further use.

#### Protective Effects of SSWE on Cobia Against *Phdd*

*P. damselae* subsp. *damselae* (*Phdd*, strain Raca-LPT) was isolated from moribund cobia at a local farm in 2019, and its identity was confirmed using Analytical Profile Index (API) 20E Test (bioMérieux, France, **Table S1**) and 16S rRNA sequence using primers 16S-F: agagtttgatcatggctcag and 16S-R: ggttaccttgttacgactt (**Figure S1**). The bacterium was grown on thiosulfate-citrate-bile salt-sucrose (TCBS) agar plates (**Figure S2**) to verify its identity from a close bacterial species *P. damselae* subsp. *piscicida* (*Phdp*). The antibiotic sensitivity test for *Phdd* was also determined using standard experimental procedures (**Table S2**). To determine the median lethal dose (LD50) of *Phdd* to cobia, 50 healthy fish (weighing 150–200 g) were divided into five groups in separated FRP tanks (1 m^3^) (N = 10 per group). Four groups were intraperitoneally challenged with *Phdd* suspensions from 3.125 × 10^6^ to 2.5 × 10^7^ CFU/g BW (see **Table S3**). The control group was injected with PBS. The volume injected was again 1% of BW. Mortality rates were recorded daily for 8 d and the LD50 was calculated based on the program of (23). To fulfill the criteria of Koch’s postulates, bacteria were isolated again from challenged moribund fish and the identity of the isolate was verified.

Fish were randomly allocated to two FRP tanks (1 m^3^) with continuous fresh seawater flow (2 L/min). Fish (average weight 150 ± 15 g) were injected intraperitoneally with PBS or PBS containing SSWE (20 μg/g BW) 8 h prior to being challenged with *Phdd* that had been cultured on tryptic soy agar (TSA; Difco, USA) supplemented with 2% NaCl for 16 h at 28°C before being washed with PBS. In the first and second trials, fish were challenged with PBS or PBS containing *Phdd* at 4.5 × 10^6^ colony forming units (CFUs)/g BW (1/2 × LD_50_, n = 10 per group) and 1.2 × 10^7^ CFU/g BW (1 × LD_50_, n = 12 per group), respectively, by peritoneal injection of the bacterial suspension 8 h post-SSWE stimulation. The survival of fish in each group was recorded during 240 h of observation. Survival rate (SR) was determined using the following formula: SR (%) = (number of surviving fish/number of fish injected) × 100.

### Interactive Gene Expression Modulation of SSWE and *Phdd* Infection

We investigated whether the administration of SSWE could regulate the expression of immune genes in cobia after encountering pathogenic bacteria. The fish (130 ± 13 g) were divided into three groups, and the challenge dose of *Phdd* was reduced to 1.5 × 10^4^ CFU/g BW to avoid early mortality before sampling. The PBS-PBS group (the first group) was intraperitoneally injected with PBS and then was mock infected by receiving PBS 8 h post-injection. The PBS-Phdd group (the second group) was intraperitoneally injected with PBS and then challenged with *Phdd* 8 h later. Sarcodia-Phdd (the third group) was intraperitoneal injected with SSWE (20 μg/g BW) 8 h prior to *Phdd* challenge. Fish were anesthetized with MS222 and immediately sacrificed for tissue harvesting. The HK and spleen tissues were sampled 2 and 24 h post bacterial challenge. Due to limited resources and as 3R policy (replace, reduce, refine) is highly encouraged nowadays, we decided to sample three fish from each group at each time point as a responsible approach to conduct animal testing. However, it is to worth to mention that useful knowledge may lost due to limited replicates that with biological variation and may have potential obstacles for the statistical analysis.

### Gene Expression Analysis

Total RNA was extracted from the primary HK leukocytes and from HK and spleen tissue samples of cobia using TheOne Reagent (Bionovas). A spectrophotometer (SpectraMax QuickDrop; Molecular Devices, USA) was used to measure the concentration and quality of total RNA. One microgram of total RNA from each sample was subjected to DNase treatment to remove potential genomic DNA (gDNA) contamination in the RNA sample using the iScript gDNA Clear cDNA Synthesis Kit (Bio-Rad, USA), following the manufacturer’s protocol. Subsequently, the treated RNA was reverse-transcribed using the iScript Reverse Transcription Supermix. The program involved one cycle at 25°C for 5 min, followed by one cycle at 46°C for 20 min. The temperature was then raised to 95°C for 1 min to finish the reaction. cDNA samples were diluted with TE buffer (10 mM Tris–HCl, 0.1 mM disodium EDTA, pH 8.0) prior to real-time polymerase chain reaction (PCR) analysis. The expression of 11 genes, including the housekeeping gene *β-actin*, was measured using primers listed in **Table 1** with StepOnePlus (Applied Biosystems). The correct amplicons of each gene were verified by examining the melting curve using the StepOnePlus Real-Time PCR System software, and product size was verified with standard procedures for gel electrophoresis. The gene expression analysis was performed as an adaptation of (28–30). Briefly, the transcript level of each gene was calculated by comparing with a 10-fold serial dilution of the corresponding purified PCR products in the same 96-well plate using the StepOnePlus Real-Time PCR System software. The concentrations of gene of interest and housekeeping gene in samples were calculated by comparing its Ct value to that of the standard curve built from the serial dilution of corresponding PCR products with known concentration using aforementioned software. The expression of each gene in the samples was first calculated in arbitrary units by dividing the marker transcript concentration by the respective *β-actin* transcript concentration and expressed as fold-change relative to the corresponding control group.

**Table 1:** Primers used in this study.

Primer name	Forward primer (5’→3’)	Reverse primer (5’→3’)	Reference
Raca-β-actin3	ACAGACTGTTCCTCCTCCCC	AAAATCCTGAGTCAAGCGCC	(24)
Raca-*IL-1β*	CAGGCAGAACAACCACTGAC	TTCCAAGTCCAGTCCTTTGG	
Raca-*IL-6*	TCTGCGACAAGTGCACTGTTG	GGTGGCTGAAAACCTTGAACTC	
Raca-*IL-8*	GTCATTGTCATTGCTGTGGTGGTGC	CCTCGCAATGAGAATTGGCAGGAATC	
Raca-*IL-10*	ACTCCATGTGCGGCTTCAG	CCTGCCACACCATGAACAGT	
Raca-*IL-12*	CGGGCAAGTGGCATCTACA	ACCATGTGAGCTGAATGCTGAT	
Raca-*TNF-α*	AACCGGCCTCTACTTTGTCT	CGCCATCACTGCAGGAGACT	
Raca-*IFN-γ*	TACCGGGAAACCCGTCTTC	TCCAAACGCCGCTCAGA	(25)
*CC1*e	ATTACAATAAGAACCCTGTGC	TCTTTCCTGGGATGGATTTG	(26)
RC*IgM*	AGACAGCCTGCAGGGAAAAG	TGTTCCTTTCCCCCAGTAGT	(27)
Raca-*IgT*	GCAATCGATGTTTCCATCAAAG	TCTCTTCATATCCTCTCTCTTC	This study

### Statistical Analysis

The expression level of each gene was normalized to the expression of *β-actin*, and log2-transformed to improve the normality of data distribution before statistical analysis (28–30). Shapiro–Wilk and Levene’s test were performed to assess the log2-transformed data for normality and homogeneity respectively using the IBM SPSS Statistics Package 22.0 (SPSS Inc.). One‐way ANOVA followed by *post-hoc* tests were performed using Tukey’s test and Games–Howell test where appropriate, with *p <*0.05 indicating significance, to compare the levels of gene expression between the treatment and control groups in the *in vitro* and *in vivo* experiments.

## Results

### Bacterial Isolation, Identification, and Characterization

In 2019, we isolated *Phdd* (strain Raca-LPT) from cage-farmed diseased cobia. We verified its identity using the API 20E test (**Table S1**) and the 16S rRNA gene sequence (**Figure S1**) from isolated pure cultures. Bacterial identity was determined by BLAST against the GenBank database, and the highest hit was to *P. damselae* (**Figure S1**). The green colonies produced by the bacteria on TCBS agar after 20 h (**Figure S2**) indicate that the isolate was *Phdd* rather than *Phdp*, as the latter cannot grow on TCBS agar.

The virulence study suggested that the LD_50_ value for *Phdd* to cobia was 4.5 × 10^6^ CFU/g BW (**Table S3**). Bacteria were successfully re-isolated from the blood and tissues (e.g., liver, spleen, and HK) of moribund fish and were considered to meet the criteria of Koch’s postulates. Additionally, the antibiotic sensitivity test revealed that the isolate was sensitive to the quinolone class of antibiotics, namely, flumequine and oxolinic acid, but resistant to amoxycillin, doxycycline, oxytetracycline, spiramycin, erythromycin, ampicillin, and lincomycin (**Table S2**).

### Effects of SSWE on Expression of Immune-Related Genes in Primary HK Cells

**Figure 1:**
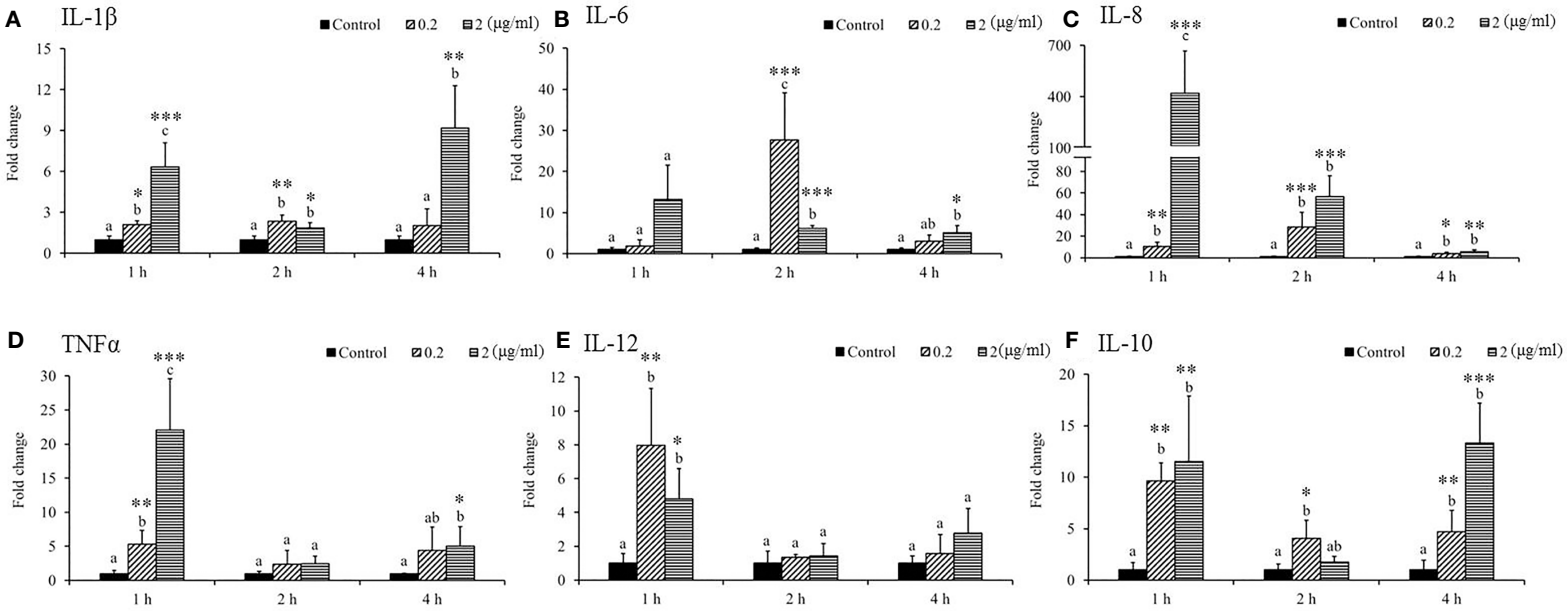
Differential immune gene expression of **(A)** *IL-1β*, **(B)** *IL-6*, **(C)** *IL-8*, **(D)** *TNFα*, **(E)** *IL-12*, and **(F)** *IL-10* in *Sarcodia suiae* water extract (SSWE) treated primary cobia head kidney (HK) cells. Cobia HK cells were stimulated with L-15 containing 0 (Control), 0.2 and 2 μg/ml of SSWE and expression of indicated genes were measured 1, 2 and 4 hours post stimulation. The expression values from three fish are shown as mean + SEM fold change relative to the control group. Significant differences between samples and their time matched control group are shown with asterisks (*p < 0.05, **p < 0.01 and ***p < 0.001) and differences between groups are indicated by different letters.

As shown in **Figure 1**, the expression of interleukin (*IL*)*-1β* significantly increased due to 0.2 and 2 μg/ml SSWE treatments, except 0.2 μg/ml SSWE at 4 h (**Figure 1A**). Expression of *IL-6* was sharply induced in both SSWE-treated groups at 2 h, and at 4 h for the group treated with 2 μg/ml SSWE (**Figure 1B**). A marked increase of *IL-8* expression was recorded for SSWE-treated groups (**Figure 1C**). *Tumor necrosis factor α* (*TNF-α*) mRNA level was upregulated in a dose-dependent manner 1 h post SSWE stimulation, and returned back to a basal level at 2 h, then elevated again for the cells treated with 2 μg/ml SSWE at 4 h (**Figure 1D**). A transient induction of *IL-12* mRNA expression was noted at 1 h post SSWE stimulation (**Figure 1E**). The transcript level of *IL-10* was significantly higher than that of the control group after stimulation with 0.2 μg/ml SSWE at all time points analyzed, while 2 μg/ml SSWE induced *IL-10* expression at 1 and 4 h (**Figure 1F**).

### SSWE Stimulated the Expression of Immune-Related Genes *In Vivo*

**Figure 2:**
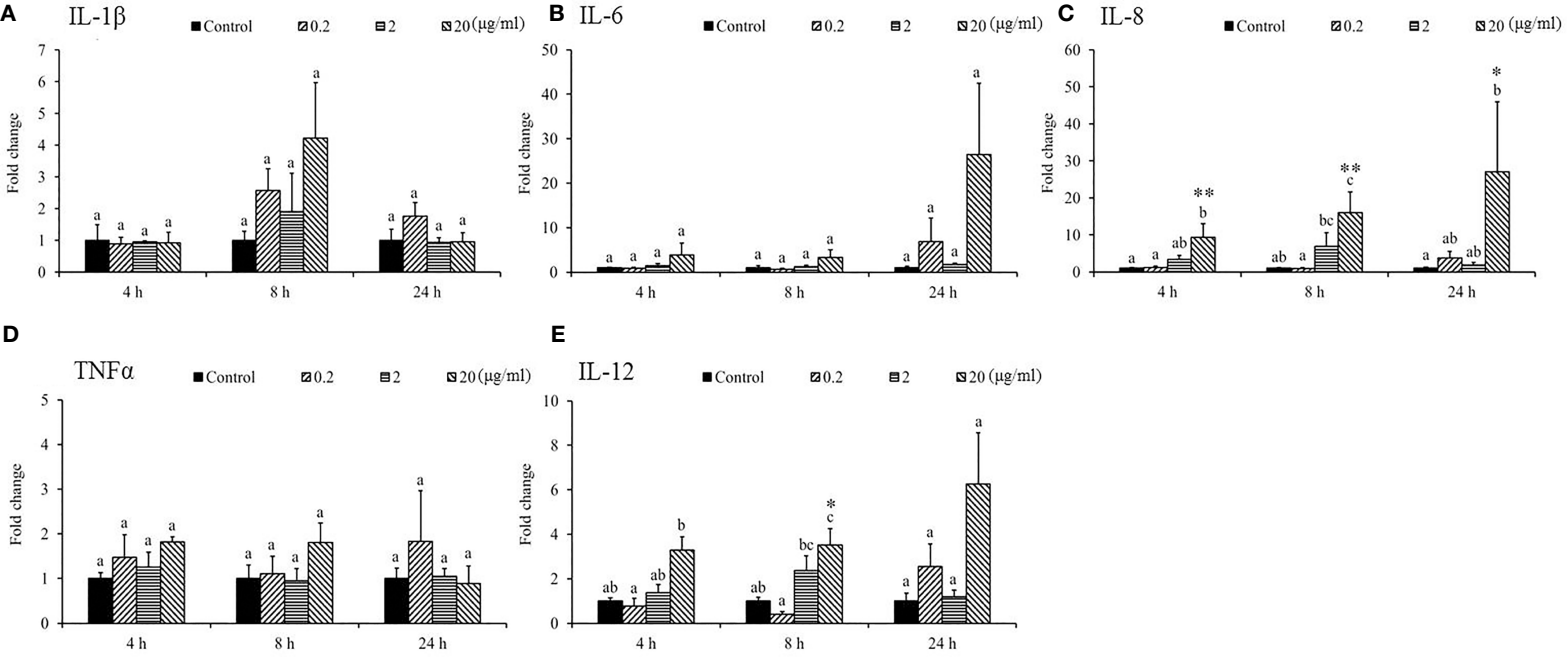
Expression of cytokine genes in the head kidney (HK) tissue from *Sarcodia suiae* water extract (SSWE) treated cobia. Fish injected intraperitoneally with PBS (Control) or SSWE were sampled and the expression of **(A)** *IL-1β*, **(B)** *IL-6*, **(C)** *IL-8*, **(D)** *TNFα*, and **(E)** *IL-12* in the HK samples was analyzed. The values are shown as mean + SEM (N = 3). Significant differences between samples and their time matched control group are shown with asterisks (*p < 0.05 and **p < 0.01) and differences between groups are indicated by different letters.

**Figure 3:**
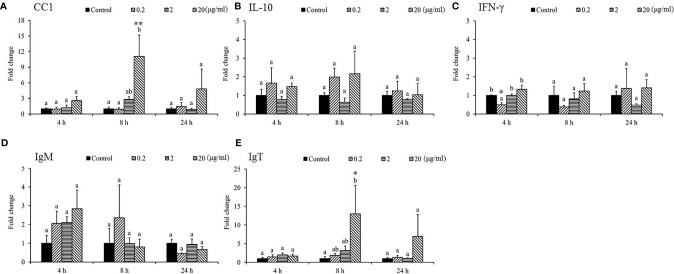
Gene expression of immune genes in the head kidney (HK) tissue from *Sarcodia suiae* water extract (SSWE) treated cobia. HK samples were collected from fish injected with PBS (Control) or SSWE and the expression of **(A)** *CC1*, **(B)** *IL-10*, **(C)** *IFN-γ*, **(D)** *IgM*, and **(E)** *IgT* was analyzed. The values are shown as mean + SEM (N = 3). Significant differences between samples and their time matched control group are shown with asterisks (*p < 0.05 and **p < 0.01) and differences between groups are indicated by different letters.

Gene expression analysis was performed to analyze the modulation of transcript levels of immune-related genes in the HK and spleen tissues after injection with various doses of SSWE to examine the effects of SSWE *in vivo*. In the HK, proinflammatory cytokines such as *IL-1β* (**Figure 2A**) and *IL-6* (**Figure 2B**) were insignificantly induced by SSWE at 4, 8, and 24 h, respectively, and *TNF-α* expression remained unaltered post-stimulation (**Figure 2D**). Interestingly, *IL-8* (**Figure 2C**) and *IL-12* (**Figure 2E**) transcript levels were elevated at 4, 8, and 24 h and 4 and 8 h respectively in the group that had the highest dose of SSWE. Expression of immune-related genes such as *IL-10* (**Figure 3B**), *interferon* (*IFN*)*-γ* (**Figure 3C**), and *immunoglobulin M* (*IgM*) (**Figure 3D**) were similar between the experimental groups and the control group post-SSWE injection, while the expressions of *CC chemokine 1* (*CC1*) (**Figure 3A**) *and IgT* (**Figure 3E**) were enhanced in the group that received 20 μg of SSWE per gram fish body weight (BW) at 8 h.

**Figure 4:**
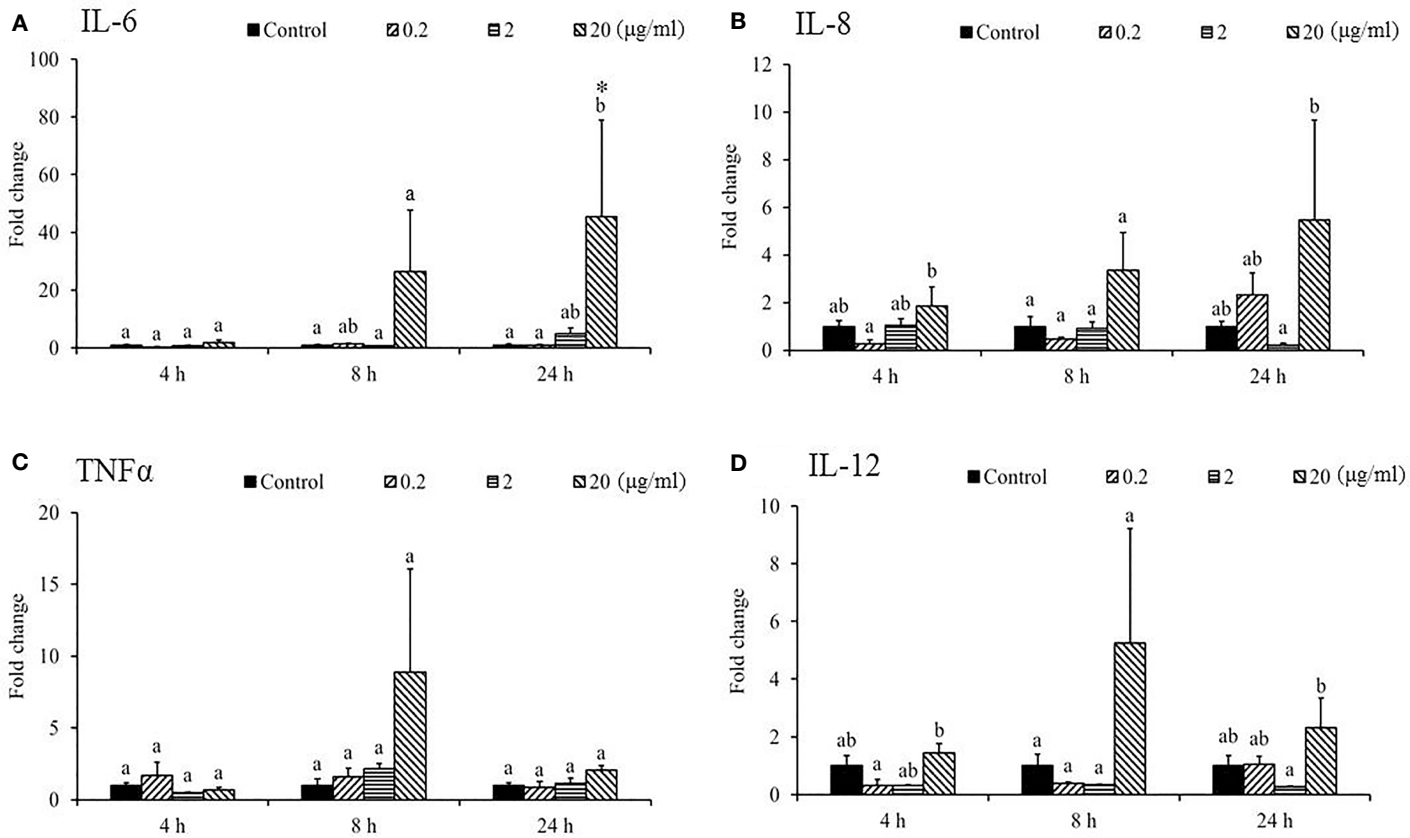
Expression of cytokine genes in the spleen tissue from *Sarcodia suiae* water extract (SSWE) treated cobia. Spleen samples were collected from fish injected with PBS (Control) or SSWE and the expression of **(A)** *IL-6*, **(B)** *IL-8*, **(C)** *TNFα*, and **(D)** *IL-12* was analyzed. The values are shown as mean + SEM (N = 3). Significant differences between samples and their time matched control group are shown with asterisks (*p < 0.05) and differences between groups are indicated by different letters.

**Figure 5:**
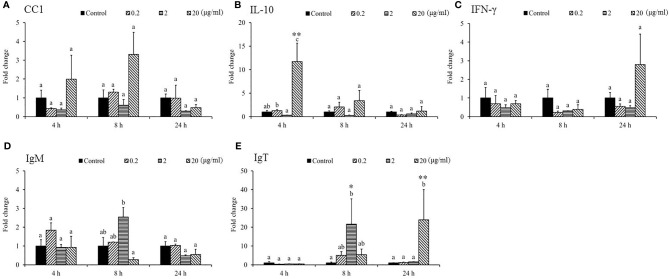
Gene expression of immune genes in the spleen tissue from *Sarcodia suiae* water extract (SSWE) treated cobia. Spleen samples were collected from fish injected with PBS (Control) or SSWE and the expression of **(A)** *CC1*, **(B)** *IL-10*, **(C)** *IFN-γ*, **(D)** *IgM*, and **(E)** *IgT* was analyzed. The values are shown as mean + SEM (N = 3). Significant differences between samples and their time matched control group are shown with asterisks (*p <0.05 and **p <0.01 and differences between groups are indicated by different letters.

The expression of *IL-1β* was under the detection limit in the spleen and was therefore omitted from the analysis. The mRNA levels of proinflammatory cytokines, namely, *IL-8* (**Figure 4B**), *TNF-α* (**Figure 4C**), *IL-12* (**Figure 4D**), *CC1* (**Figure 5A**), *IFN-γ* (**Figure 5C**), and *IgM* (**Figure 5D**) in the spleen post-SSWE treatment were upregulated, but not significantly so. Expression of *IL-6* increased over time (**Figure 4A**) while *IL-10* (**Figure 5B**) was sharply induced at 4 h in the group given the highest amount of SSWE. Noticeably, an upregulation of *IgT* was recorded in the groups that received 2 and 20 μg of SSWE per gram fish BW at 8 and 24 h, respectively (**Figure 5E**).

### SSWE Delayed Mortality During *Phdd* Infection

**Figure 6:**
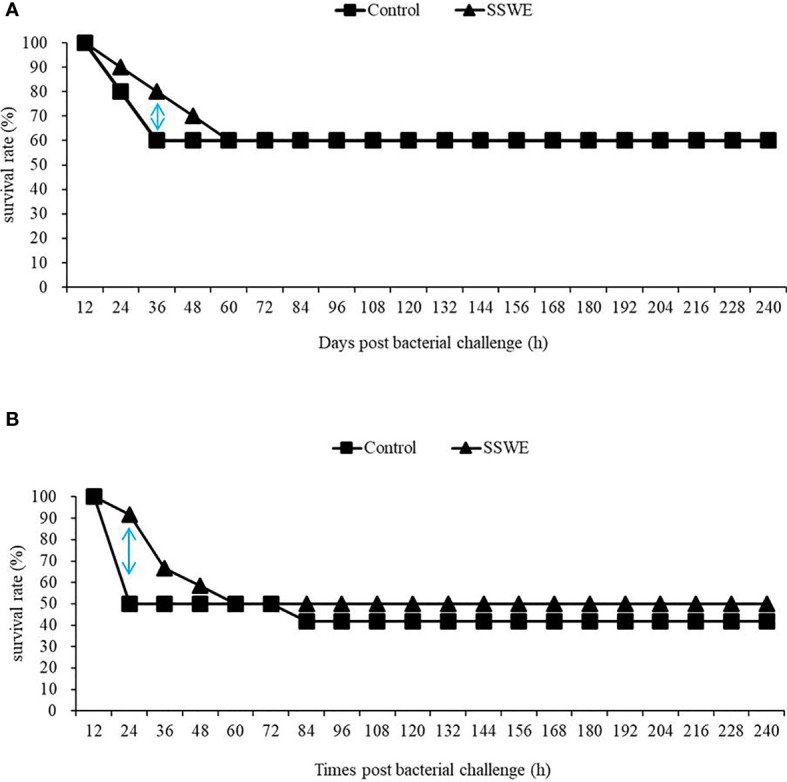
Fish were injected with PBS or PBS containing *Sarcodia suiae* water extract (SSWE, 20 μg/g body weight (BW)) followed by challenge with **(A)** 4.5 × 10^6^ CFU/g BW or **(B)** 1.2 × 10^7^ CFU/g BW *Photobacterium damselae* subsp. *damselae* 8 h post SSWE injection. Survival percentage was recorded for 240 h N = 10 **(A)** or 12 **(B)** per group.

In our previous study (20), we demonstrated that injection of Nile tilapia with SSWE prior to *Streptococcus agalactiae* challenge increased the survival rate (70%) compared to the control group (40%). In the present study, we examined the immunoprotective efficacy of SSWE in cobia against *Phdd* by conducting two challenge trials followed by SSWE treatment. The dose was set to 20 μg of SSWE per gram fish BW as it promoted higher expressions of immune-related genes in the HK and spleen compared with other dose levels. Two doses of *Phdd* (1/2 × LD_50_ and 1 × LD_50_) were used in the first and second challenge trials. Compared to the control group, the survival rate of the group that received SSWE prior to *Phdd* challenge was the same for 1/2 LD_50_ (**Figure 6A**) and was slightly higher for LD_50_ (**Figure 6B**). However, the mortality rate was clearly lower for the SSWE-treated group than the PBS-treated group during the first 24–36 h of bacterial challenge.

#### SSWE Promoted the Expression of Proinflammatory and Th1-Type Cytokines During *Phdd* Infection

**Figure 7:**
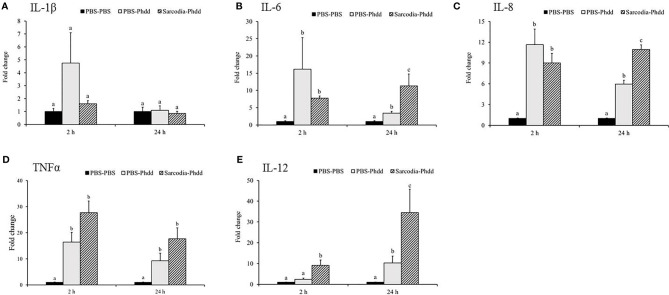
Expression of cytokine genes in the head kidney (HK) tissue from fish intraperitoneal injected with PBS and *Sarcodia suiae* water extract after challenge with *Photobacterium damselae* subsp. *damselae* (*Phdd*). The relative expression profile of **(A)** *IL-1β*, **(B)** *IL-6*, **(C)** *IL-8*, **(D)** *TNFα*, and **(E)** *IL-12* in the HK samples was analyzed. The values are shown as mean + SEM (N = 3). Significant differences (*p < 0.05*) are indicated by different letters.

**Figure 8:**
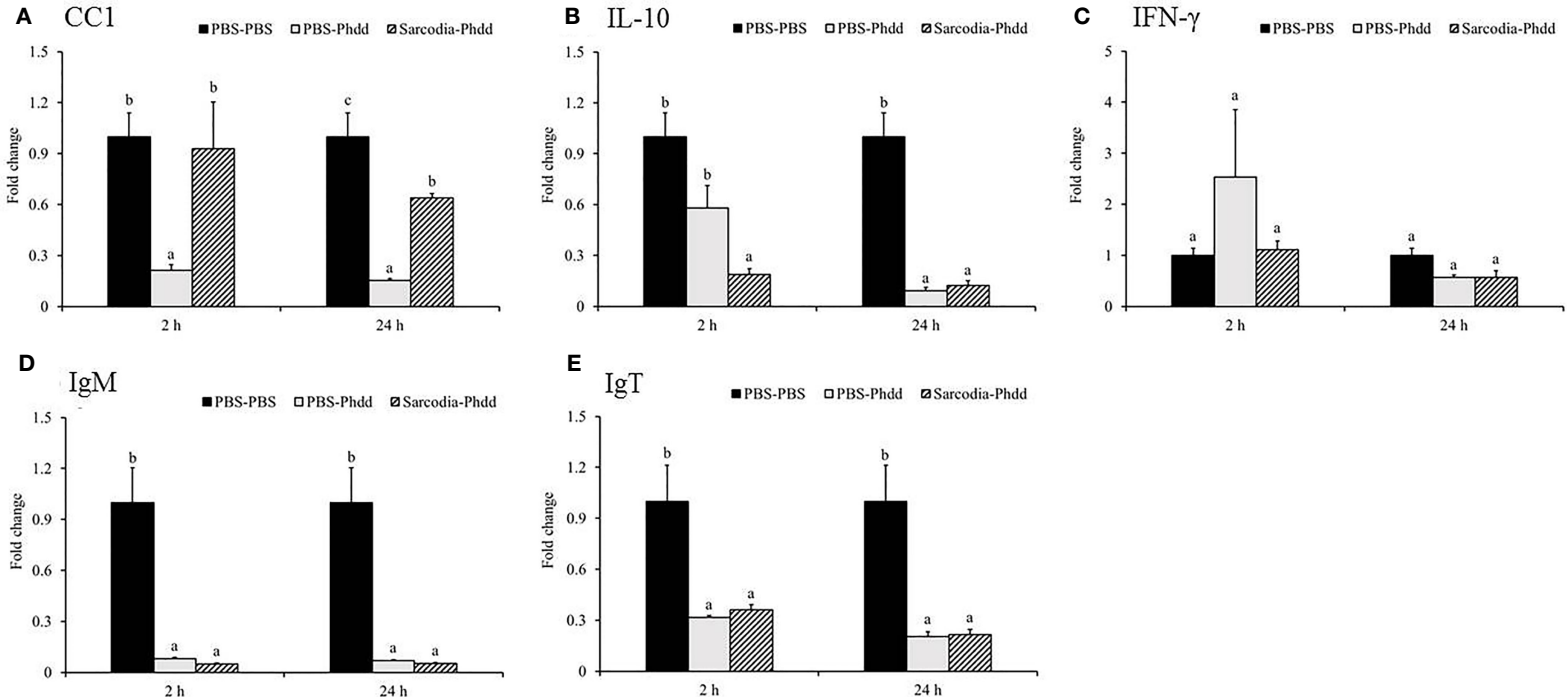
Expression of immune genes in the head kidney (HK) tissue from fish intraperitoneal injected with PBS and *Sarcodia suiae* water extract after challenge with *Photobacterium damselae* subsp. *damselae* (*Phdd*). The relative expression profile of **(A)** *CC1*, **(B)** *IL-10*, **(C)** *IFN-γ*, **(D)** *IgM*, and **(E)** *IgT* in the HK samples was analyzed. The values are shown as mean + SEM (N = 3). Significant differences (*p < 0.05*) are indicated by different letters.

We investigated potential mechanisms to explain how SSWE delays mortality in cobia during *Phdd* infection. We conducted a similar trial with a lower dose of bacteria to avoid early mortality, then analyzed the expression of immune-related genes in the HK and spleen tissues. In the HK, expressions of *IL-1β* (**Figure 7A**) and *IFN-γ* (**Figure 8C**) were not different among the experimental groups. *IL-6* (**Figure 7B**), *IL-8* (**Figure 7C**), and *IL-12* (**Figure 7E**) were induced by *Phdd* infection and were higher in the Sarcodia-Phdd group than the other two groups at 24 h post infection. The transcript level of *TNF-α* (**Figure 7D**) was elevated in the HK post bacterial challenge but was not different between PBS-Phdd and Sarcodia-Phdd. The expression of *IL-10* (**Figure 8B**), *IgM* (**Figure 8D**), and *IgT* (**Figure 8E**) were significantly downregulated in the HK of PBS-Phdd and Sarcodia-Phdd compared to PBS-PBS. Compared to PBS-PBS, the *CC1* transcript level was downregulated for PBS-Phdd at 2 and 24 h, and was downregulated for Sarcodia-Phdd at 24 h (**Figure 8A**).

**Figure 9:**
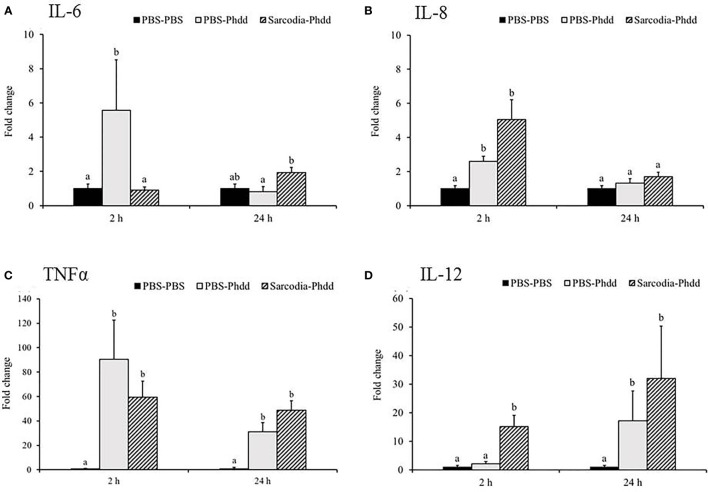
Comparison of transcript level of cytokine genes in the spleen tissue of fish intraperitoneal injected with PBS and *Sarcodia suiae* water extract after challenge with *Photobacterium damselae* subsp. *damselae* (*Phdd*). The relative expression profile of **(A)** *IL-6*, **(B)** *IL-8*, **(C)** *TNFα*, and **(D)** *IL-12* in the spleen samples was analyzed. The values are shown as mean + SEM (N = 3). Significant differences (*p < 0.05*) are indicated by different letters.

**Figure 10:**
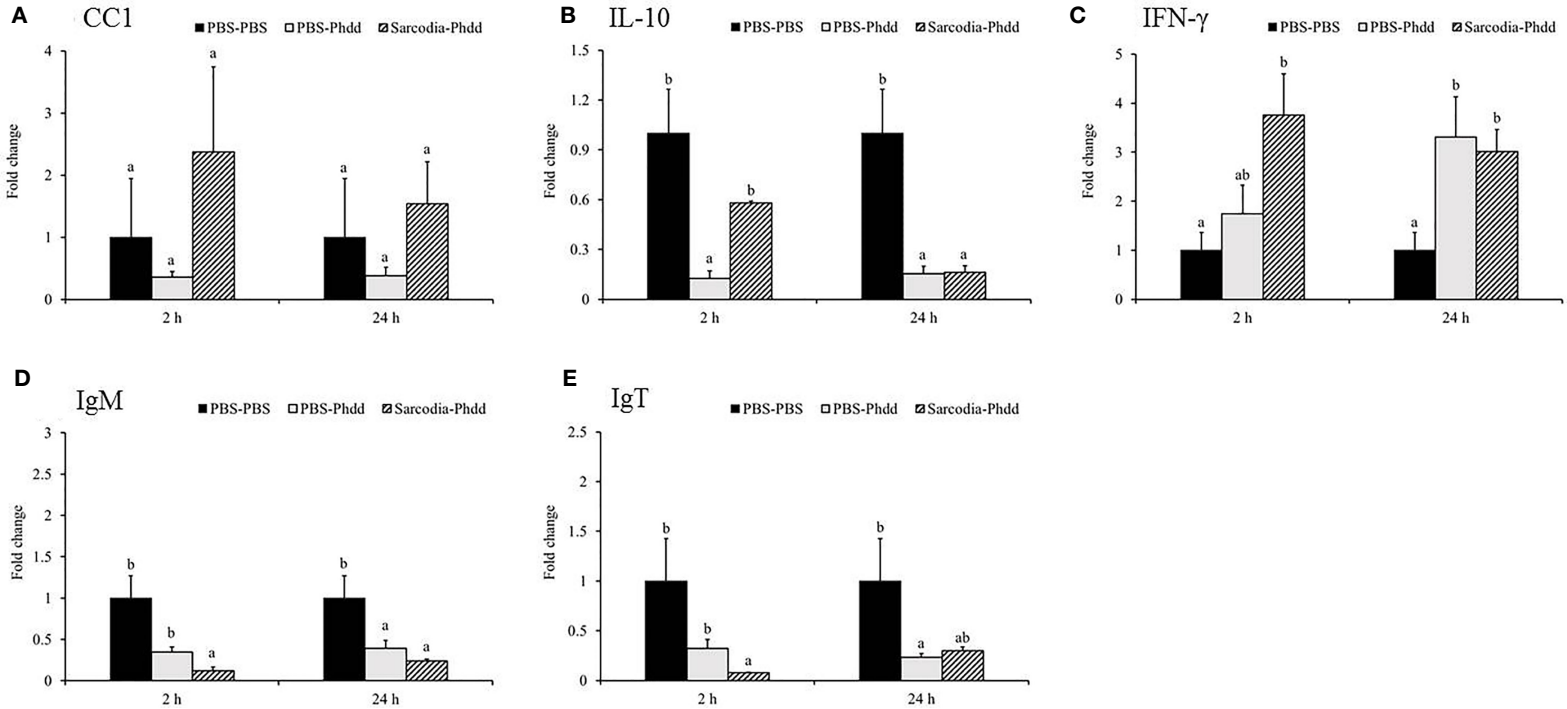
Comparison of transcript level of immune genes in the spleen tissue of fish intraperitoneal injected with PBS and *Sarcodia suiae* water extract after challenge with *Photobacterium damselae* subsp. *damselae* (*Phdd*). The relative expression profile of **(A)** *CC1*, **(B)** *IL-10*, **(C)** *IFN-γ*, **(D)** *IgM*, and **(E)** *IgT* in the spleen samples was analyzed. The values are shown as mean + SEM (N = 3). Significant differences (*p < 0.05*) are indicated by different letters.

In the spleen, *IL-6* (**Figure 9A**) was upregulated for PBS-Phdd 2 h post-challenge and was unaltered for Sarcodia-Phdd. Elevated expressions of *IL-8* (**Figure 9B**) and *TNF-α* (**Figure 9C**) were observed in the challenged groups but were not enhanced further by SSWE treatment. In contrast, expressions of *IL-12* (**Figure 9D**) and *IFN-γ* (**Figure 10C**) were noticeably higher for Sarcodia-Phdd than the other two groups at 2 h post-challenge. *CC1* expression (**Figure 10A**) was not significantly altered in the challenged groups, while *IL-10* (**Figure 10B**), *IgM* (**Figure 10D**), and *IgT* (**Figure 10E**) were lower in the spleen for PBS-Phdd and Sarcodia-Phdd than the PBS-PBS control group.

## Discussion

The fast-growing nature of cobia has made it one of the most popular fish species in marine aquaculture, especially in the United States and other Asian regions. Since the early 1990s, Taiwan has been breeding and growing cobia in Pingtung County and Penghu, but disease due to bacteria and parasites often cause substantial mortality and major economic damage.

There are two subspecies of *P. damselae*, namely, *P. damselae* subsp. *damselae* (*Phdd*) and *P. damselae* subsp. *piscicida* (*Phdp*). Harmful effects of *Phdp* on cobia have often been reported (14), while *Phdd* infections in cobia were first reported in 2016 (31). In October 2019, a pathogenic bacterium was isolated from diseased cage-cultured cobia, and its identity was confirmed as *Phdd* (**Figures S1, S2**). Similar to a previous study (31), the outbreak occurred in the summer at higher water temperatures (31 ± 0.6°C), and the diseased fish had hepatic and renal hemorrhages (**Figure S3**). However, the LD_50_ value was higher in our current study (1.2 × 10^7^ CFU/g BW) than the previous study (4.1 × 10^5^ CFU/g BW) (31), which may have resulted from the larger size of fish in the current work (fish average weight 150 ± 15 g compared to a mean weight of 20 g).

Marine-derived polysaccharides (MDPs)—such as fucoidan, carrageenan, galactan, agar, ulvan, alginate, laminarin, chitin, and chitosan—have been shown to be useful for promoting the immune status of fish and shellfish (32). In addition to assessing the effects of SSWE on cobia, we aimed to understand the regulation of immune genes in cobia after encountering *Phdd* and the interactive gene expression modulation by the application of SSWE and *Phdd* infection.

Sulfated polysaccharides of *Sargassum angustifolium* increase the release of IL-1β, IL-6, IL-10, IL-12, and TNF-α in RAW264.7 macrophages (33). Similarly, sulfated polysaccharides isolated from green algae (*Codium fragile*) enhance the expression of *IL-8* and *TNF-α* in the primary HK cells of olive flounder (*Paralichthys olivaceus*) (34). IL-1β is a proinflammatory cytokine that induces a series of inflammatory responses to enable organisms to quickly respond to infections (35). It is an important mediator in inflammatory responses in fish (36, 37). IL-6 is a proinflammatory cytokine that can induce the development and differentiation of lymphocytes, cell proliferation, cell survival, and apoptosis signals (38). TNF-α is a proinflammatory cytokine that plays an important role in a variety of host responses (39). TNF-α also regulates the production of other inflammatory cytokines and enhances the function of a variety of cellular responses (40). SSWE comprises sulfated polysaccharides (20), and we showed that *in vitro* SSWE treatment increased the expression of proinflammatory cytokines in primary HK leukocytes, which suggests SSWE has immunoenhancing effects in cobia. We conducted an *in vivo* trial to further assess its biological effects in cobia by examining the expression modulation of immune-related genes in the HK and spleen after injection with various doses of SSWE. As with limited capacity and resources, replicated tanks were not adopted in the *in vivo* trials. However, fish for the experimental groups were allocated into identical (or very similar) FRP tanks side by side with the same water source, light and aeration system. We therefore consider the tank specific effects should be minor.

Intraperitoneal injection cobia with SSWE (20 μg/g BW) induced the expression of proinflammatory cytokines (e.g., *IL-6* and *IL-12*) in the HK and spleen, which may due to the activation of immune cells such as macrophages, B cells and T cells in the peritoneal cavity. Similarly, Jin et al. reported that injection of fucoidan significantly increased the expression of *IL-6* in the spleen of mice (41) and higher *IL-6* expression was also found in the spleen of *Solea senegalensis* injected with sulfated polysaccharide extracted from *Ulva ohnoi* prior to *Phdp* challenge (42). Likewise, *IL-6* expression was induced after infection, and fish that received SSWE had higher transcript levels of *IL-6* in the HK. Immediate production of IL-12 by macrophages after infection/stimulation is regarded indispensable for activation of a protective immune response against pathogens (43). IL-12 induces the immune response of Th1 cells (44), while Th1 cells secrete IFN-γ, TNF-α, IL-2, and TNF-β, which are essential for the elimination of intracellular pathogens (45). Th2 cells produce cytokines IL-4, IL-5, IL-6, IL-10, and IL-13 (46) for antibody production and elimination of extracellular microorganisms (45). We showed that the transcript levels of *IL-12*, *TNF-α*, and *IFN-γ* were often higher in the HK and spleen in Sarcodia-Phdd than PBS-Phdd. Additionally, expressions of Th2-related factors such as *IL-10* in the HK, and *IgM* and *IgT* in the spleen were lower for fish that received SSWE instead of PBS before *Phdd* challenge. IL-10 is known to inhibit the activation of Th1 cells and the production of related cytokines, and is also a major inhibitor of the immune response and other factors (47). The suppressed expression of *IL-10* revealed the antagonistic modulation of pro- and anti-inflammatory genes. Moreover, the activation of immune cells by SSWE rendered elevated expression of proinflammatory and Th1 cytokines that may contribute to the delayed mortality of cobia against *Phdd* infection. Such phenomenon was also reported in *S. senegalensis* that injected with sulfated polysaccharides from *U. ohnoi* followed by challenge with *Phdp* (42). This indicates that SSWE treatment facilitated the activation of Th1-like responses in cobia to fight against *Phdd* infection, widen the window of opportunity for treatments, and may be valuable as an immunostimulant or vaccine adjuvant for fish.

IL-8 (also called CXCL8) is an important chemokine related to the proinflammatory process (48) while CC chemokines are involved in inducing the migration of monocytes and other cell types, and the reactions of antibacterial and antiviral immune responses in fish (26, 49). *O. niloticus* CC1 (On-CC1) has been shown to be involved in the early immune responses to bacterial pathogens and recombinant rOn-CC1 protein efficiently enhanced the phagocytic activity of phagocytes (50). The expression of *CC1* was elevated by SSWE treatment. Interestingly, *Phdd* infection resulted in suppressed expression of *CC1* in the HK, suggesting *Phdd* may manipulate the immune responses of the host by interfering with the recruitment of immune cells. This suppression of *CC1* expression was not seen in the Sarcodia-Phdd group, indicating that SSWE treatment can abolish the suppressive effects of *Phdd*. IL-12 can protect the host against parasites, viruses, and intracellular bacteria by stimulating natural killer cells and T cells to produce IFN-γ (51). Intraperitoneal injection of SSWE led to enhanced expression of *IL-8* and *IL-12* in the HK of cobia, as was similarly observed in Nile tilapia (20). Expression of *IL-8* and *IL-12* were upregulated post-bacterial challenge, and were sustained in the HK of the Sarcodia-Phdd group and higher than the PBS-Phdd group 24 h post infection. This implies that the SSWE treatment maintained proinflammatory responses in cobia against *Phdd*. IFN-γ can induce antimicrobial and antitumor mechanisms, upregulate antigen processing and antigen presentation pathways, and enhance the activities of natural killer cells (52) and B cells (53, 54). A previous study has shown that mice infected with *Escherichia coli* or *Listeria monocytogenes* had elevated IFN-γ production in splenic innate B cells, which promoted an innate response against these bacteria (55). The expression of *IFN-γ* in the spleen or HK were not enhanced by intraperitoneal injection of sulfated polysaccharides from green algae in *Paralichthys olivaceus* (34) or SSWE in cobia, but was significantly upregulated in the spleen of the Sarcodia-Phdd group after *Phdd* infection. This indicates that the immune system can distinguish harmful pathogens (e.g., bacteria) from non-harmful immunostimulants (e.g., algal polysaccharides), and promote the activation of different effectors.

## Conclusions

Overall, the water extract of *S. suiae* acts as an immunostimulant. Injection of SSWE (20 μg/g body weight) promotes the expression of pro-inflammatory cytokines in the immune-related organs of cobia, and delay the onset of death during *Phdd* infections. Application of SSWE facilitated the activation of Th1-like responses in cobia to fight against *Phdd* infection. The potential use of SSWE as an immunostimulant or vaccine adjuvant for fish in aquaculture is worth exploring.

## Data Availability Statement

The original contributions presented in the study are included in the article/**Supplementary Material**. Further inquiries can be directed to the corresponding authors.

## Ethics Statement

The animal study was reviewed and approved by the NTOU Institutional Animal Care and Use Committee.

## Author Contributions

P-TL designed the study. The study was supervised by F-HN, C-CT, and M-CL. P-YC conducted the qPCR study and challenge trials. P-TL wrote the first version of the manuscript. All authors contributed to the article and approved the submitted version.

## Funding

This work was funded by the Council of Agriculture, Executive Yuan, Republic of China (Taiwan) under grant agreement no. 109AS-9.3.4-FA-F1.

## Conflict of Interest

The authors declare that the research was conducted in the absence of any commercial or financial relationships that could be construed as a potential conflict of interest.

## Publisher’s Note

All claims expressed in this article are solely those of the authors and do not necessarily represent those of their affiliated organizations, or those of the publisher, the editors and the reviewers. Any product that may be evaluated in this article, or claim that may be made by its manufacturer, is not guaranteed or endorsed by the publisher.
