# Supplementary material for: Sarcodia suiae Water Extract Promotes the Expression of Proinflammatory and Th1-Type Cytokines and Delay the Onset of Mortality in Cobia (Rachycentron canadum) During Photobacterium damselae subsp. damselae Infection
Source: Front Immunol. 2022 Jan 24;12:801501. doi: 10.3389/fimmu.2021.801501 (PMC8820276; doi:10.3389/fimmu.2021.801501)
Supplement: Supplementary file 1 [file DataSheet_1.docx]

Supplementary Material

Table S1. Biochemical characteristics of *Photobacterium damselae* subsp. *damselae* used in the present study using API 20E system

| Presence of |  | |
| --- | --- | --- |
| β-galactosidase (ONPG) | - | |
| Arginine dihydrolase (ADH) | + | |
| Lysine decarboxylase (LDC) | + | |
| Ornithine decarboxylase (ODC) | - | |
| Urease (URE) | + | |
| Tryptophan deaminase (TDA) | - | |
| Gelatinase (GEL) | - | |
| Production of | |  |
| H_2_S | - | |
| Indole (IND) | - | |
| Acetoin (VP) | - | |
| fermentation of | |  |
| Citrate (CIT) | - | |
| Glucose (GLU) | + | |
| Mannitol (MAN) | - | |
| Inositol (INO) | - | |
| Sorbitol (SOR) | - | |
| Rhamnose (RHA) | - | |
| Sucrose (SAC) | - | |
| Melibiose (MEL) | - | |
| Amygdaline (AMY) | - | |
| Arabinose (ARA) | - | |

Table S2. Antibiotic sensitivity test of *Photobacterium damselae* subsp. *damselae* used in the present study

| Antibiotic | Disc content (μg) | Strain inhibition zone (mm) | Sensitivity |
| --- | --- | --- | --- |
| Amoxycillin | 30 | 9 | R |
| Doxycycline | 30 | 6 | R |
| Oxytetracycline | 30 | 6 | R |
| Flumequine | 30 | 23 | S |
| Spiramycin | 100 | 9 | R |
| Erythromycin | 15 | 9 | R |
| Oxolinic acid | 2 | 20 | S |
| Ampicillin | 10 | 6 | R |
| Lincomycin | 2 | 9 | R |

*S: Susceptible; R: Resistant

**Table S3. Mortality of cobia challenged with *Photobacterium damselae* subsp. *damselae***

| Bacterial dose (CFU) | Cumulative mortality (%) | LD50 |
| --- | --- | --- |
| 2.50 × 10^7^ CFU/g BW | 80% | 1.25 × 10^7^ CFU/g BW |
| 1.25 × 10^7^ CFU/g BW | 50% |  |
| 6.25 × 10^6^ CFU/g BW | 20% |  |
| 3.125 × 10^6^ CFU/g BW | 10% |  |
| Control (PBS) | 0% |  |

CFU, colony forming unit; LD50, median lethal dose; BW, body weight


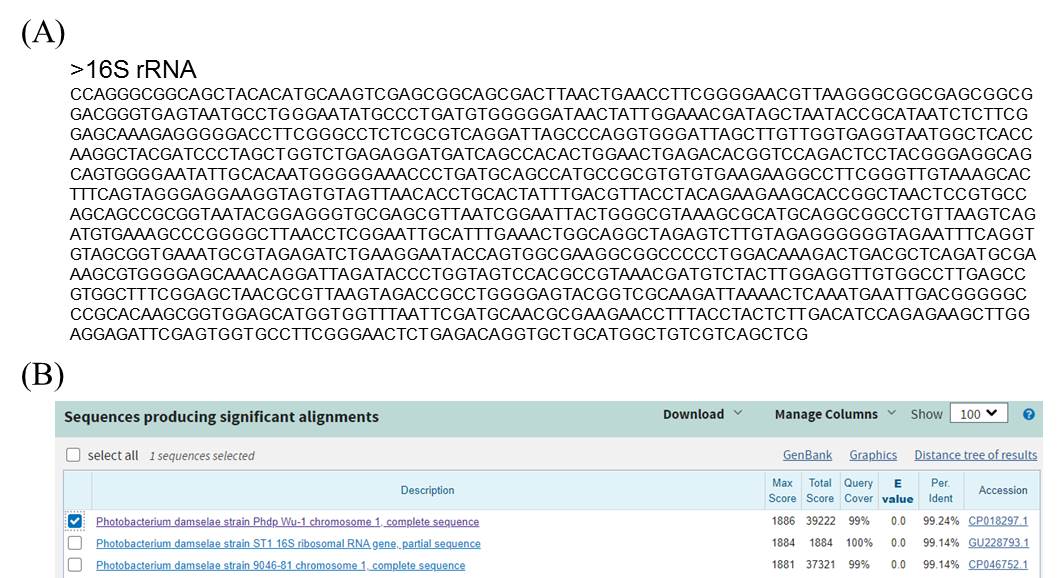


Fig. S1. (A) Sequencing results of amplicons amplified from 16S rRNA gene from *Photobacterium damselae* subsp. *damselae* and (B) BLAST results obtained using NCBI blastn.


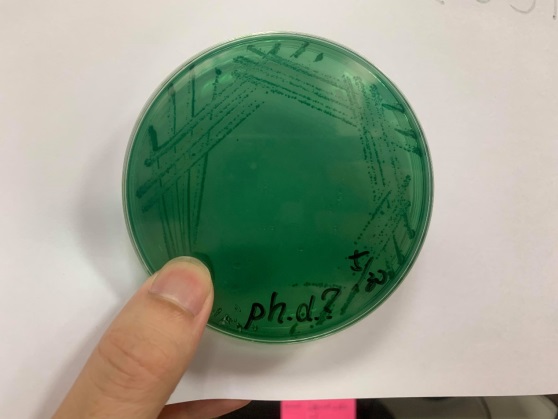


Fig. S2. Growth of *Photobacterium damselae* subsp. *damselae* on TCBS agar.


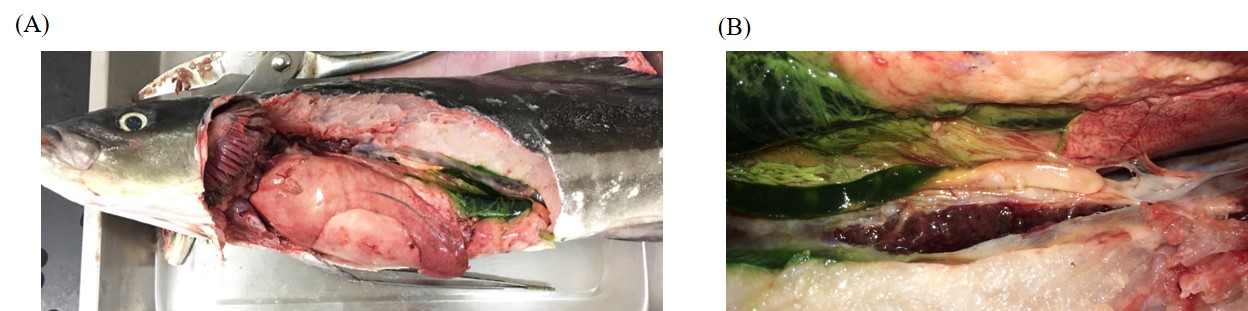


Fig. S3. *Photobacterium damselae* subsp. *damselae* infected cobia displaying haemorrhaging in the (A) liver and (B) kidney.
